# Meteotsunamis in the Laurentian Great Lakes

**DOI:** 10.1038/srep37832

**Published:** 2016-11-24

**Authors:** Adam J. Bechle, Chin H. Wu, David A. R. Kristovich, Eric J. Anderson, David J. Schwab, Alexander B. Rabinovich

**Affiliations:** 1 Department of Civil and Environmental Engineering, University of Wisconsin-Madison, Madison, WI, USA; 2 Climate and Atmospheric Science Section, ISWS, Prairie Research Institute, University of Illinois at Urbana–Champaign, Urbana, Illinois, USA; 3 National Oceanic and Atmospheric Administration, Great Lakes Environmental Research Laboratory, 4840 S. State Rd, Ann Arbor, MI 48108, USA; 4 Water Center, University of Michigan, Ann Arbor, MI, USA; 5 Fisheries and Oceans Canada, Institute of Ocean Sciences, 9860 W. Saanich Rd., Sidney, BC, Canada; 6 P.P. Shirshov Institute of Oceanology, Russian Academy of Sciences, 36 Nakhimovsky Pr., Moscow, Russia

## Abstract

The generation mechanism of meteotsunamis, which are meteorologically induced water waves with spatial/temporal characteristics and behavior similar to seismic tsunamis, is poorly understood. We quantify meteotsunamis in terms of seasonality, causes, and occurrence frequency through the analysis of long-term water level records in the Laurentian Great Lakes. The majority of the observed meteotsunamis happen from late-spring to mid-summer and are associated primarily with convective storms. Meteotsunami events of potentially dangerous magnitude (height > 0.3 m) occur an average of 106 times per year throughout the region. These results reveal that meteotsunamis are much more frequent than follow from historic anecdotal reports. Future climate scenarios over the United States show a likely increase in the number of days favorable to severe convective storm formation over the Great Lakes, particularly in the spring season. This would suggest that the convectively associated meteotsunamis in these regions may experience an increase in occurrence frequency or a temporal shift in occurrence to earlier in the warm season. To date, meteotsunamis in the area of the Great Lakes have been an overlooked hazard.

Meteotsunamis are meteorologically generated water waves that have temporal and spatial characteristics similar to seismic tsunamis and can pose serious hazards to coastal communities^1^. Meteotsunami waves, which typically have periods from 2 mins to 2 hours^2^, have caused disastrous effects to property and life along coasts worldwide due to their significant runup and strong associated currents^3^,^4^,^5^,^6^,^7^,^8^. Even meteotsunamis with seemingly modest heights (~0.3 m) can produce dangerous currents^9^,^10^ that have created hazardous conditions for recreational users^11^. Episodic analysis of large events has revealed that meteotsunamis are mainly caused by atmospheric pressure and wind perturbations associated with frontal passages^12^, cyclones^13^,^14^, atmospheric gravity waves^15^, and mesoscale convective systems^16^,^17^, including derechos^18^. Atmospheric energy is constantly fed into the wave if the propagation speed of the atmospheric disturbance is approximately equal to the local free wave speed, which is dependent upon water depth for open-ocean long waves^19^ and shelf slope for coastally trapped edge waves^20^. Heights of meteotsunami can further increase at the coast through local mechanisms such as shoaling, shelf resonance, reflection, and harbor resonance^11^,^21^,^22^,^23^,^24^. Owing to the ubiquity of atmospheric disturbances in pressure and wind, meteotsunamis can add to risk posed by seismic tsunamis^25^ or present a threat to regions which are not traditionally recognized as under risk of seismic tsunamis^26^. Nevertheless, to date a quantitative assessment of meteotsunamis occurrence frequency and risk is limited^27^,^28^.

The Laurentian Great Lakes, which form the Earth’s largest freshwater system and include over 10,000 miles of coastline, are an example of a region with low seismic activity but a long history of impactful meteotsunami events, as illustrated in Fig. 1 4,11,18,29,30,31,32 (*see* supplemental Table S1 *for a list of historic events*). The disastrous effects of meteotsunamis in the Great Lakes were most vividly demonstrated in 1954, when a three meter meteotsunami killed seven people in Chicago, IL, USA^4^. Recently, notable meteotsunamis have occurred in 2012, when three swimmers required rescue after being swept a kilometer offshore into Lake Erie near Cleveland, OH, USA^11^ and in 2014, when a Lake Superior meteotsunami overtopped the Soo Locks, interrupted shipping operations, and prompted homes to be evacuated in Saulte Ste. Marie, ON, Canada. Most of the reported Great Lakes meteotsunamis have occurred near densely populated areas (Fig. 1), possibly due to a reporting bias towards population centers. Furthermore, wave transformations such as reflection in the enclosed basins can result in a meteotsunami wave that is decoupled from the causative atmospheric disturbance^11^,^21^, which not only leads to increased risk for coastal communities but can also cause misidentification of the source of these waves. As a result, a credible quantitative assessment of meteotsunami occurrence in the Great Lakes has been lacking until recently when the occurrence of meteotsunamis was quantified for the first-time using 20-year historic water level records at 10 sites around Lake Michigan^28^. Nevertheless, the occurrence frequency of meteotsunamis on a regional scale such as the entire Great Lakes region has yet to be assessed and the risk posed by these coastal hazards remains unclear.

The objective of this paper is to characterize the occurrence frequency of meteotsunamis in the Great Lakes, as well as the timing of and causative storms associated with these events. Long-term water level and radar records across the region are analyzed to quantify meteotsunami return levels, associated storm structure types, and monthly and annual occurrence distributions. Patterns in Great Lakes-wide regional meteotsunami characteristics are then analyzed in the context of the geographic, bathymetric, and atmospheric settings of the region. This is the first study that provides insight into the occurrence frequency of meteotsunamis throughout the Laurentian Great Lakes region, as well as the mechanisms which influence the regional meteotsunami characteristics.

## Results

To reveal meteotsunami occurrence in all five Great Lakes, we first identified meteotsunamis from water level records of up to 20 years at 32 locations across the region. Specifically, meteotsunami events are defined in this study as water level oscillations with period less than 2 hours and a height above 0.3 m that are associated with strong surface wind and/or barometric pressure perturbations at a nearby weather station^1^. Of the identified high-frequency water level oscillations which exceed 0.3 m, approximately 87% are associated with strong atmospheric perturbations and thus deemed to be meteotsunami. Those oscillations which are not related to atmospheric perturbations are typically either apparent gauge errors which feature a single instantaneous change in water level or reflected meteotsunami waves that have already struck that station at an earlier time.

**Figure 1:**
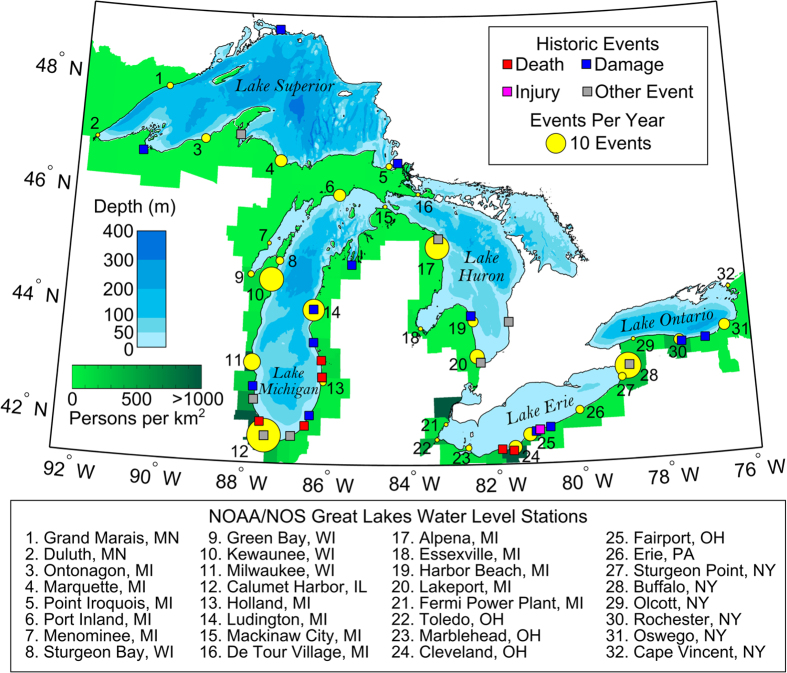
Historic meteotsunami events in the Great Lakes. Map of meteotsunamis reported in the Great Lakes with bathymetry contours and adjacent county-level population density. In addition, the average number of meteotsunamis observed at each station per year is plotted as a circle scaled by area. Figure was created using MATLAB-2016 edition (http: http://www.mathworks.com/) with bathymetry data from NOAA Centers for Environmental Information and population data from United States Census Bureau.

The average number of meteotsunamis observed per year at each station in the Great Lakes is illustrated in Fig. 1. Calumet Harbor, IL in Lake Michigan experiences the greatest number of meteotsunamis (29 per year), followed by Buffalo, NY in Lake Erie (17 per year) and Alpena, MI in Lake Huron (14 per year). On a lake-by-lake basis, Lake Michigan experiences the highest frequency of meteotsunami occurrence, at 51 events per year, followed by Lakes Erie (27 events), Huron (17 events), Superior (6 events), and Ontario (5 events). Overall, an average of 106 meteotsunami events occurred per year throughout the entire Great Lakes region. To date, little is known about the occurrence of meteotsunamis in the world. In this study, more events were identified per year in the water level records than were reported in the entire historical literature since 1882, suggesting that meteotsunamis with potentially hazardous magnitude occur much more frequently in the Great Lakes than had been previously expected.

**Figure 2:**
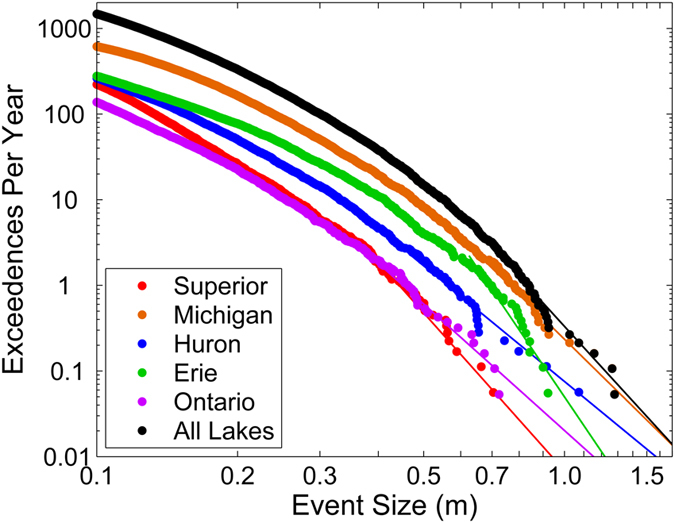
Meteotsunami size-frequency distributions for each lake. Lake-wide meteotsunami height observations (dots) fit with the Pareto Type 1 distribution (solid line) for each lake.

To calculate the occurrence frequency of extreme meteotsunamis, a Peaks Over Threshold (POT) approach was taken to fit the Pareto Type 1 distribution to the meteotsunami size data in each lake^33^,^34^. The resulting distributions are plotted in Fig. 2, with an annual meteotsunami magnitude (i.e. 1 exceedance per year) for the Great Lakes of 0.83 m and a 10-year (i.e. 0.1 exceedances per year) return level of 1.3 m. For reference, the largest recorded water level oscillation observed during the deadly 1954 Chicago meteotsunami was approximately 1 m^35^, a return level which occurs throughout the Great Lakes with a recurrence interval of 3 years (i.e. 0.33 exceedance per year). Thus, while meteotsunamis of this magnitude were previously thought of as a rare phenomenon, water level analysis reveals that large meteotsunamis are rather regular in the Great Lakes. Nevertheless, water level oscillations associated with meteotsunamis are not considered in planning or design along the Great Lakes coasts, nor can current forecasting systems predict their occurrence for public safety efforts^11^. In general, the meteotsunami threat is a serious coastal hazard for the Great Lakes that has to date been underestimated.

**Figure 3:**
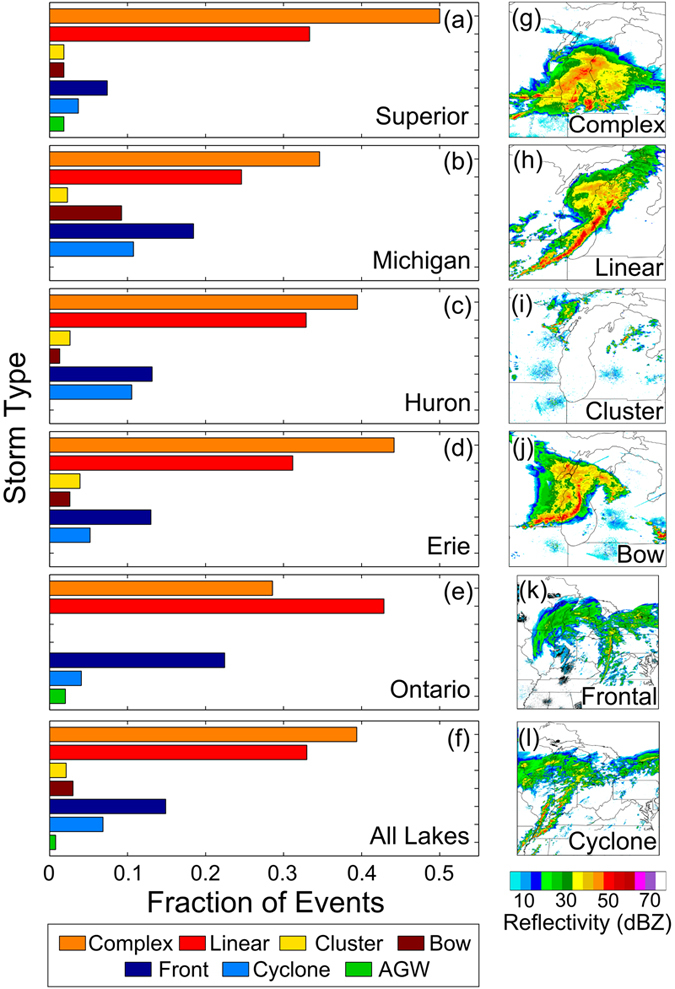
Storm structures associated with meteotsunamis. (**a**)–(**e**) Distribution of storm structures associated with meteotsunamis which exceed the 1-year return level in each lake and (**f**) for all lakes combined. Storm structures are illustrated in sample radar reflectivity images for meteotsunamis observed in Lake Michigan: (**g**) convective complex, (**h**) linear convection, (**i**) convective cluster, (**j**) bow convection, (**k**) frontal – note the center of low pressure is greater than 200 km from Lake Michigan, and (**l**) cyclone – note the center of low pressure is over Lake Michigan. Note that quasi-circular light-blue features in panels i and j are non-meteorological targets seen close to the radar sites. Figure was created using MATLAB-2016 edition (http://www.mathworks.com/) with radar data from the Iowa Environmental Mesonet NEXRAD Composite database.

To examine the origin of meteotsunamis in the Great Lakes, the storm types associated with identified events which exceed the one-year return level height are classified from radar reflectivity imagery. Storms are classified as one of seven categories^36^,^37^,^38^,^39^: convective cluster, convective complex, linear convection, bow convection (including derechos), extratropical cyclone, frontal (i.e. fronts associated with distant extratropical cyclones), and possible atmospheric gravity waves (AGW), defined in this study as strong pressure or wind perturbations in the absence of an associated storm. Across the entire Great Lakes, convective-type storms are associated with 78% of the meteotsunamis. Complex (39%) and linear (33%) convective storm structures are the two most common storm types associated with meteotsunamis (Fig. 3a–f). Convective complexes are associated with the greatest number of meteotsunamis in each lake except for Lake Ontario, where linear convective structures dominate. For non-convective events, the fraction of meteotsunamis associated with extratropical cyclone fronts is the greatest in Lake Ontario, whereas Lake Michigan and Lake Huron experience the greatest fraction of events associated with extratropical cyclones. Pressure and wind perturbations in without a nearby storm, which are possibly related to AGW, comprise a small fraction of the observed meteotsunamis. This suggests that if atmospheric gravity waves do play an important role in Great Lakes meteotsunamis, they are usually associated with convective or frontal storms and were not distinguished from the storms in this study. Overall, our analysis reveals that meteotsunamis in the Great Lakes are primarily associated with complex and linear convective storm structures, with a secondary contribution from extratropical cyclone-type structures.

**Figure 4:**
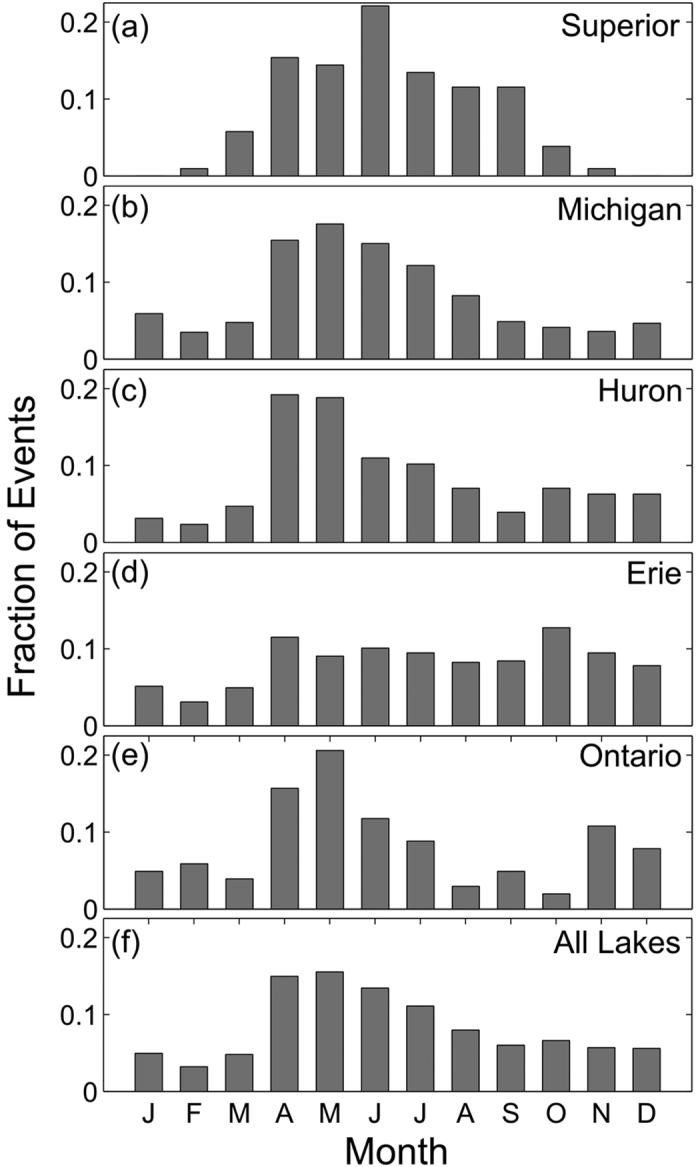
Monthly time of meteotsunami occurrence. (**a**)–(**e**) Monthly distributions of meteotsunamis observed in each lake and (**f**) for all lakes combined.

**Figure 5:**
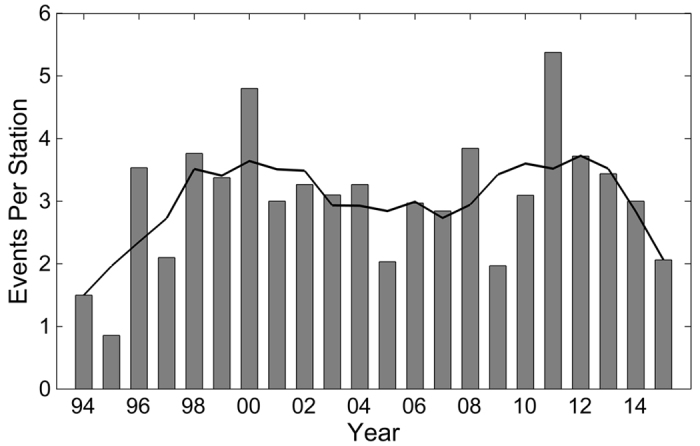
Annual distribution of meteotsunami events in the Great Lakes. The distribution is normalized for the number of active stations in a given year. The black line indicates a five-year moving average.

To estimate when meteotsunamis are most likely to arise, monthly and annual distributions of meteotsunamis have been computed. The peak time of meteotsunami events within the year is calculated as the circular mean month of the observed events. Figure 4a–f shows the monthly distribution of meteotsunamis in the Great Lakes. Lakes Michigan and Huron have similar annual monthly distributions, with a circular mean month of occurrence of 5.5 and 5.4 months, respectively. Meteotsunami season in Lake Ontario is earlier in the spring (4.7 months), whereas Lake Superior (6.2 months) meteotsunamis appear slightly later in the summer. Interestingly, Lake Erie, by far the shallowest Great Lake, exhibits a prolonged meteotsunami season that extends from late spring to late fall, with a late summer circular mean month of occurrence (7.9 months). Aggregated throughout the entire region (Fig. 4f), meteotsunami occurrences rise sharply in April, reach a maximum in May, and gradually decrease in frequency until October, yielding a circular mean month of meteotsunami occurrence in late May (circular mean of 5.8 months). The annual distribution of meteotsunami events per station operating in a given year is illustrated in Fig. 5, with a five-year moving average of annual occurrence also plotted to visualize longer-term trends. For the Great Lakes as a whole, there is a bimodal distribution in annual occurrences with local maxima in five-year moving average at 2000 and 2012 and a local minimum at 2007. Further study of the observed oscillatory pattern in annual occurrence may provide insight into the role of large-scale climate processes on meteotsunami occurrence.

## Discussion

For the first time, long-term water level records throughout multiple water bodies in a region (e.g. the Laurentian Great Lakes) are analyzed to identify spatial and temporal patterns in meteotsunami occurrence. The results reveal that meteotsunamis in the Great Lakes are much more common phenomenon than anecdotal historical reports indicate. Great Lakes meteotsunamis are associated primarily with linear and complex convective storms and generally come about from the late-spring to mid-summer. This meteotsunami timing coincides with beginning of the summer swimming and recreation season, putting many lake users at risk to the danger posed by meteotsunami. These findings suggesting that to date meteotsunamis have been an underrated hazard for the Great Lakes region.

In the following, meteotsunami occurrence statistics are viewed on a regional scale to reveal patterns in frequency, causative storms, and seasonality, which are then compared with the physical and atmospheric setting of the region. In terms of meteotsunami occurrence frequency, water level stations which experience the greatest number of meteotsunamis per year tend to be located towards the southwest portion of the Great Lakes. This observed pattern is also true on a lake-by-lake basis (Fig. 2), where Lake Michigan experiences the most frequent and largest meteotsunamis, followed by Lakes Huron and Erie. These patterns in meteotsunami occurrence frequency are consistent with the overall distribution of observed strong convective thunderstorms in the region, which are most frequent in the southwest Great Lakes region^40^. This finding indicates that meteotsunamis tend to be most frequent in areas where convective storms are most frequent.

The distribution of specific storm structures is also consistent with climatological patterns in storm distribution. Convective complexes are associated with the greatest number of meteotsunamis in each lake except for Lake Ontario, where linear convective structures dominate. These results are similar to the spatial distribution of mesoscale convective complexes in the region, which varies longitudinally across the Great Lakes with peak activity in the west^41^. For non-convective events, the fraction of meteotsunamis associated with extratropical cyclone fronts is the greatest in Lake Ontario, which has been observed to experience the largest density of frontal passages among the five lakes^42^. Both Lake Michigan and Lake Huron experience the greatest fraction of events associated with extratropical cyclones, consistent with spatial peaks in the occurrence frequency of strong cyclones over the Great Lakes^43^.

Interestingly, while meteotsunamis in the Great Lakes are associated primarily with convective storms, the observed peak time of meteotsunamis in the Great Lakes precedes that of the mid-summer peak in convective activity^40^. The late-spring to early summer meteotsunami seasonality in the Great Lakes corresponds well with the incidence of the strongly-stable overlake boundary layer^39^,^44^, though a mechanistic connection of boundary layer physics to meteotsunami formation was unclear^28^. Another possible explanation for the discontinuity between meteotsunami and storm season is seasonal variations in storm speeds. Cloud level winds (850 hPa to 300 hPa), which are positively related to convective storm speed^45^, tend to be greatest in the Great Lakes region early in the year and decrease to a minimum in the mid-summer, according to observed upper air sounding climatologies complied by the NOAA Storm Prediction Center. It is known that faster storm propagation speeds are required to excite propagation resonance of edge waves over steeper bottom slopes^20^ and open-ocean waves over deeper water depths^19^. In short, while convective activity peaks in the mid-summer, faster storm speeds in the late-spring and early summer may be more conducive to propagation resonance with lake bathymetry than slower storm speeds in the mid-summer.

Under a changing climate, the patterns observed here in meteotsunami occurrence frequency, cause, and season may shift. The contribution of cyclones to Great Lakes meteotsunamis may decrease in the future, as the frequency of tropical and extratropical cyclones are projected to decrease under a changing climate^46^. On the other hand, simulations of future climate scenarios over the United States show a likely increase in the number of days favorable to severe convective storm formation over the Great Lakes, particularly in the spring season^47^,^48^,^49^. This would suggest that the convectively associated meteotsunamis in these regions may experience an increase in occurrence frequency or a temporal shift in occurrence to earlier in the warm season. Upper air wind speeds have been observed to increase over North America^50^, which may lead to more frequent storm speeds favorable to meteotsunami production and a shift of meteotsunami season. Overall, meteotsunamis may become even more frequent under a changing climate. To date, coastal hazards and risks induced by meteotsunamis in the Great Lakes have been overlooked!

## Methods

Long-term water level data are obtained from 32 monitoring stations operated on the Great Lakes by the National Oceanic and Atmospheric Administration (NOAA) National Ocean Service (NOS), with gauge locations illustrated by yellow circles in Fig. 1. At each station, 6-minute water level data with an average time series length of 17 years have been recorded. Water level data from Canada are not included in the study owing to limited record length at high-frequency temporal resolution. Nevertheless, the NOAA/NOS data analyzed in this study spans the major axis of each lake, yielding a representative approximation of meteotsunami occurrence throughout the lake. The water level time series are high-pass filtered with a cutoff-period of 6 hours and individual waves with period less than 2 hours and height greater than 0.3 m are identified as possible meteotsunamis^1^. These possible meteotsunamis are then cross-referenced with surface meteorological records obtained from nearby National Weather Service (NWS) Automated Surface Observing System (ASOS) stations to determine if the oscillations were associated with perturbations in barometric pressure or wind speed. An events is considered a meteotsunami if nearby meteorological records indicate either a pressure perturbation with temporal gradient in excess of 0.15 hPa/min (e.g. a 0.75 hPa change over 5 minutes) or wind speed in excess of 10 m/s, thresholds based on analysis of meteotsunamis in Lake Michigan^51^, which are consistent with observations worldwide^52^,^53^,^54^. To account for the possibility of reflected meteotsunami waves^11^,^21^, an atmospheric perturbation is considered to be related to a meteotsunami wave if it occurred within the time period required for an open-water long wave to travel across the long axis of a lake (with wave celerity based on average depth), ranging from 3 hours for Lake Ontario to 8 hours for Lake Erie. If no atmospheric perturbations occurred before the initiation of the water level oscillations within this lake-dependent time period, the wave was deemed to be not directly meteorologically generated and was removed from further analysis. Multiple water level oscillations that meet these meteotsunami criteria within the same 12 h period are consolidated to a single event represented by the largest wave.

A Peaks Over Threshold (POT) approach of extreme value statistics is used to represent extreme meteotsunami size-frequency data in each lake^28^. First, the meteotsunami wave heights at all stations in each lake are aggregated. To address the condition that a single meteotsunami impacts multiple stations, meteotsunamis that occurred in the same lake within the same 12 hour period are considered a single event represented by the largest observed wave^28^. Meteotsunami events are also combined over all five lakes into an “All Lakes” data set. The data are fit to the Pareto Type 1 (PT1) distribution^33^,^34^ which is commonly fit to seismic tsunami height statistics^34^ and is described by the shape parameter *β* and a local height threshold *x*_*m*_. To establish *x*_*m*_, a failure-to-reject method is employed by sorting the height observations and deleting the lowest values until the distribution is no longer rejected by the one-sided Anderson-Darling goodness of fit test (*α* = 0.1)^55^. The shape parameter *β* is estimated with the Maximum Likelihood Estimate. Meteotsunami event probabilities are represented in terms of mean recurrence intervals (RI), the inverse of which express the probability that specified return level (RL) magnitudes will be exceeded in any one year. The PT1 distribution was chosen among other extreme value distributions such as the Generalize Extreme Value, Weibull, Power, and Generalized Pareto Distributions based on the Anderson-Darling goodness of fit tests as well as the stability of parameter estimates during the failure-to-reject threshold selection process^33^. Though the PT1 does exhibit power law-type unbounded growth in return levels, a likelihood ratio test for Lake Michigan meteotsunami size data^28^ failed to reject (α = 0.05) the PT1 in favor of the Generalized Pareto Distribution, which contains the PT1 as a special case and can have bounded growth. Owing to the unbounded growth of the PT1, we are careful not to consider recurrence intervals extrapolated beyond the length of the data record. Challenges associated with event catalogs composed from multiple locations are recognized^34^ and these results are used only to examine meteotsunami magnitude differences between the lakes.

NEXRAD base reflectivity radar imagery made available by the Iowa Environmental Mesonet at 5 minute intervals is used to classify the structure of storms associated with meteotsunami events greater than the 1 year return level at each station. Storms are classified as one of seven categories: convective cluster, convective complex, linear convection, bow convection, extratropical cyclone, frontal, or atmospheric gravity wave (AGW)^36^,^37^,^38^,^39^. Detailed descriptions of the criteria used to classify storm structures are available in^28^. For clarity, we define possible AGW here as strong pressure or wind perturbations in the absence of a convective or frontal storm, conditions which have been observed in previous studies of meteotsunamis^56^. Though atmospheric gravity waves may also occur with convective or frontal storms^52^,^54^, further detailed analysis would be needed to determine the relative importance of atmospheric gravity waves within these parent storms^57^, which was beyond the scope of this study. A sample NEXRAD radar image is provided in Fig. 3(g–l) for each storm category except AGW since the criteria used in this study for AGW do not require appreciable radar-indicated features.

The monthly and annual timing of meteotsunami occurrences is analyzed on a lake-by-lake basis. The meteotsunami events are binned into histograms by both month and year of occurrence. The monthly distributions of meteotsunami occurrence are expressed as the fraction of events in each lake normalized by the total number of observed meteotsunamis in the lake. The annual distributions of meteotsunami occurrence are expressed as the number of observed event per year normalized by the number of active water level stations in each year.

## Additional Information

**How to cite this article**: Bechle, A. J. *et al*. Meteotsunamis in the Laurentian Great Lakes. *Sci. Rep.* **6**, 37832; doi: 10.1038/srep37832 (2016).

**Publisher's note:** Springer Nature remains neutral with regard to jurisdictional claims in published maps and institutional affiliations.

## Supplementary Material

Supplementary Information
