# Supplementary material for: Meteotsunamis in the Laurentian Great Lakes
Source: Sci Rep. 2016 Nov 24;6:37832. doi: 10.1038/srep37832 (PMC5121654; doi:10.1038/srep37832)
Supplement: Supplementary Information [file srep37832-s1.pdf]

Supplemental Information for

Meteotsunamis in the Laurentian Great Lakes

Adam J. Bechle[1], Chin H. Wu[1*], David A.R. Kristovich[2], Eric J. Anderson[3], David J. Schwab[4], and Alexander B. Rabinovich[5,6]

[1]Department of Civil and Environmental Engineering, University of Wisconsin-Madison, Madison, WI

[2]Climate and Atmospheric Science Section, ISWS, Prairie Research Institute, University of Illinois at Urbana–Champaign, Urbana, Illinois

[3]National Oceanic and Atmospheric Administration, Great Lakes Environmental Research Laboratory, 4840 S. State Rd, Ann Arbor, MI 48108

[4]Water Center, University of Michigan, Ann Arbor, MI

[5]Fisheries and Oceans Canada, Institute of Ocean Sciences, 9860 W. Saanich Rd., Sidney, BC, Canada

[6]P.P. Shirshov Institute of Oceanology, Russian Academy of Sciences, 36 Nakhimovsky Pr., Moscow, Russia.

Summary

Supporting Table S1 documents the reported meteotsunami events in the Great Lakes were used to create Figure 1 (historic events). Events were aggregated from published literature and unpublished reports. Unpublished reports of meteotsunamis were collected from a survey of newspaper databases, web search engines, and social media (Twitter, YouTube) using keywords “meteotsunami”, “meteo-tsunami”, “meteorological tsunami”, “seiche”, “tidal wave”, and “tsunami”.

Table S1. Historic Great Lakes meteotsunamis from literature and news reports.

Lake	Location	Date	Height (m)	Damage	Injuries	Deaths	Reference
Superior	Ashland, WI	14 September 1895	2.0	Y			A tidal wave swept over Lake Superior [1895]
Superior	Rossport, ON	3 October 1933	-	Y			Lake port damaged [1933]
Superior	Keweenaw Bay, MI	31 May 2011	1.0				jblehman [2011]
Superior	Sault St. Marie	4 September 2014	1.0	Y			Meyers [2014]
Michigan	St. Joseph, MI	7 April 1983	1.5	Y			Mortimer [2004]
Michigan	Chicago, IL	26 June 1954	3.0			7	Ewing et al. [1954]
Michigan	Michigan City, IN	26 June 1954	1.0				Harris [1957]
Michigan	Calumet Harbor	31 May 1998	1.7				As-Salek and Schwab [2004]
Michigan	White Lake, MI	31 May 1998	1.7	Y			As-Salek and Schwab [2004]
Michigan	Waukegan, IL	3 August 1960	1.5				Donn and Ewing [1956]
Michigan	Grand Haven, MI	4 July 1929	6.0			10	Fifth body recovered from lake today [1929]
Michigan	Holland, MI	13 July 1938	3.0			3	Reynolds [2001]
Michigan	Warren Dunes, IN	4 July 2003	1.5			7	Guenther [2003]
Michigan	Kenosha, WI	12 May 1912	1.5	Y			Tidal wave caused great damage on west shore of Lake Michigan [1905]
Michigan	Ludington, MI	1 July 1956	3.0	Y			Wind, water on rampage [1956]
Michigan	Traverse City, MI	10 June 2015	-	Y			Torregrossa [2015]
Huron	Presque Isle, MI	29 June 2012	3.0				Sepic and Rabinovich [2014]

Huron	Harbor Beach, MI	23 May 1925	1.5	Y			Weather states acrobatic stunt [1925]
Huron	Goderich, ON	5 May 1952	0.5				Donn [1959]
Huron	Fort Gratiot, MI	22 August 1971	1.0				Murty and Freeman [1973]
Erie	Cleveland, OH	22 August 1971	4.0	Y	Y	1	Murty and Freeman [1973]
Erie	Ashtabula, OH	23 June 1882	-	Y			A tidal wave sweeps the lake front [1882]
Erie	Cleveland, OH	12 April 1912	5.0			7	Tidal wave sweeps Erie [1912]
Erie	Cleveland, OH	31 May 1942	0.5				Freak tidal wave sweeps Lake Erie shoreline [1942]
Erie	Buffalo, NY	5 May 1952	1.0				Donn [1959]
Erie	Madison, OH	5 May 1952	2.0		Y		Donn [1959]
Erie	Perry, OH	27 May 2012	-	Y			Anderson et al. [2015]
Ontario	Sodus Point, NY	27 May 2012	1.3	Y			Anderson et al. [2015]
Ontario	Rochester, NY	23 May 1925	1.5	Y			Tidal wave rolled up along shores of Lake Ontario [1925]
Ontario	Greece, NY	7 August 1979	1.3				Chaston [1979]

References

Anderson, E.J., Bechle, A.J., Wu, C.H., Schwab, D.J., Mann, G.E., and Lombardy, K.A. (2015), Reconstruction of a meteotsunami in Lake Erie on 27 May 2012: Roles of atmospheric conditions on hydrodynamic response in enclosed basins. *J. Geophys. Res. Oceans*. 120, 8020–8038, doi: 10.1002/2015JC010883.

As-Salek, J.A., and D.J. Schwab (2004), High-frequency water level fluctuations in Lake Michigan, Journal of Waterway, Port, *Coastal and Ocean Engineering*, 130(1), 45-53. doi: 10.1061/(ASCE)0733-950X.

Chaston, P.R. (1979) An unusual weather phenomenon: The Rochester Seiche, *Weatherwise*, 32(5), 211.

Donn, W. L. (1959), The Great Lakes storm surge of May 5, 1952, *J. Geophys. Res*., 64(2), 191–198, doi:10.1029/JZ064i002p00191.

Donn, W.L., and M. Ewing (1956), Stokes' edge waves in Lake Michigan, *Science*, 124, 1238–1242, doi: 10.1126/science.124.3234.1238.

Ewing, M., F. Press, and W. J. Donn (1954), An explanation of the Lake Michigan wave of 26 June 1954, *Science*, 120, 684–686, doi:10.1126/science.120.3122.684.

Fifth body recovered from lake today (1929, July 5), *Grand Haven Daily Tribune*.

Freak Tidal Wave Sweeps Lake Erie Shoreline; Eight Believed Drowned (1942, May 31), *Toledo Blade*.

Guenther, D. (2003) Rip current case study 3, 4 July 2003, report, Marquette Michigan National Weather Service Office, Marquette, MI.

Harris DL (1957), The effect of a moving pressure disturbance on the water level in a lake, *Meteor. Mon.*, 2(10), 46-57.

jblehman, (2011, May 31), *Lake Superior seiche* [Video file], Retrieved from https://www.youtube.com/watch?v=bYI1zIjJr4g&feature=youtu.be .

Lake Port Damaged – Strange tidal wave carries away fishing docks (1933, October 4), *The Montreal Gazette*.

Meyers, J. (2014, September 5), *Lake Superior water floods part of Sault Ste. Marie*, http://www.duluthnewstribune.com/content/lake-superior-water-floods-part-sault-ste-marie

Mortimer, C.H. (2004), Lake Michigan in Motion, University of Wisconsin Press, Madison, WI, pp 304.

Murty, T. S., and N.G. Freeman (1973), Applications of the concepts of edge waves and numerical modelling to storm surge studies on Lake Huron, in Proc., 16th Conf. Great Lakes Research, pp. 533-548, Intl. Association of Great Lakes Research, Ann Arbor, Michigan.

Reynolds, G.D. (2001), Fatal wave: The “Seiche”, The Joint Archives of Holland, 10(2), 1-4.

Šepić, J., and A. B. Rabinovich (2014), Meteotsunami in the Great Lakes, Chesapeake Bay and on the Atlantic coast of the United States generated by the propagating 'derecho' of 29–30 June 2012, *Nat. Hazards*, 74, 75–107, doi:10.1007/s11069-014-1310-5.

Strange water phenomenon on Lake Ontario (2012, July 9), retrieved from http://13wham.com/news/local/story/lake-ontario-water-seiche/oe3hbk9e6emrrwpw4ffglg.cspx?rss=102.

Tidal wave caused great damage on west shore of Lake Michigan (1905, May 12), *Sandusky Star Journal*.

Tidal wave rolled up along shores of Lake Ontario (1925, May 25), *The Montreal Gazette*.

A tidal wave sweeps the lake front doing considerable damage – docks four feet under water – hundreds of fish washed ashore (1882, June 24), *Cleveland Plain Dealer*.

Tidal wave sweeps Erie (1912, April 14), *New York Times*.

A tidal wave swept over Lake Superior (1895, September 21), *The Quebec Saturday Budget*.

Torregrossa, M. (2015, June 10), *Great Lakes 'tidal wave' near Traverse City caused by U.P. storms*, retrieved from http://www.mlive.com/weather/index.ssf/2015/06/great_lakes_tidal_wave_near_tr.html

Wind, water on rampage (1956, July 2), *Ludington Daily News*.

Weather stages acrobatic stunt (1925, May 24), *Ludington Sunday Morning News*.
